# Large Outbreaks of Ciguatera after Consumption of Brown Marbled Grouper

**DOI:** 10.3390/toxins6072041

**Published:** 2014-07-11

**Authors:** Thomas Y. K. Chan

**Affiliations:** 1 Division of Clinical Pharmacology, Department of Medicine and Therapeutics, Faculty of Medicine, the Chinese University of Hong Kong, Prince of Wales Hospital, Shatin, New Territories, Hong Kong, China; E-Mail: tykchan@cuhk.edu.hk; Tel.: +852-2632-3907; Fax: +852-2646-8756; 2 Centre for Food and Drug Safety, Faculty of Medicine, the Chinese University of Hong Kong, Hong Kong, China

**Keywords:** ciguatera, ciguatoxins, brown marbled grouper, *Epinephelus fuscoguttatus*

## Abstract

Brown marbled grouper (*Epinephelus fuscoguttatus*) is an apex predator from coral reefs of the Indo-Pacific region. All five published case series of ciguatera after consumption of brown marbled grouper were reviewed to characterize the types, severity and chronicity of ciguatera symptoms associated with its consumption. Three of these case series were from large outbreaks affecting over 100–200 subjects who had eaten this reef fish served at banquets. Affected subjects generally developed a combination of gastrointestinal, neurological and, less commonly, cardiovascular symptoms. Gastrointestinal symptoms occurred early and generally subsided in 1–2 days. Some neurological symptoms (e.g., paresthesia of four limbs) could last for weeks or months. Sinus bradycardia and hypotension occurred early, but could be severe and prolonged, necessitating the timely use of intravenous fluids, atropine and dopamine. Other cardiovascular and neurological features included atrial ectopics, ventricular ectopics, dyspnea, chest tightness, PR interval >0.2 s, ST segment changes, polymyositis and coma. Concomitant alcohol consumption was associated with a much higher risk of developing bradycardia, hypotension and altered skin sensation. The public should realize that consumption of the high-risk fish (especially the ciguatoxin-rich parts and together with alcohol use) and repeated ciguatoxin exposures will result in more severe and chronic illness.

## 1. Introduction

Ciguatera results from eating large, predatory tropical and subtropical coral reef fishes that have bioaccumulated ciguatoxins (CTX) [1,2]. These lipid-soluble and heat-stable toxins originate from dinoflagellates of the genus *Gambierdiscus* species [3]. Biotransformation and bioaccumulation occur when CTX pass through the food chain [1], such that large, predatory reef fishes are more likely to be toxic [4]. Large outbreaks of ciguatera mostly occur in discrete regions of the Pacific Ocean, Indian Ocean and Caribbean Sea, between latitudes 35°N and 35°S. However, with increases in international tourism and coral reef fish trade and consumption [4], ciguatera constitutes a public health problem in all parts of the world. Affected individuals typically present with a constellation of gastrointestinal, neurological, cardiovascular and other signs and symptoms [1]. The predominant clinical features and the types, severity and duration of symptoms vary with individual susceptibility, geographical region, type and dose of the CTX involved and the origin of ciguatoxic fish [1,2,5].

**Figure 1:**
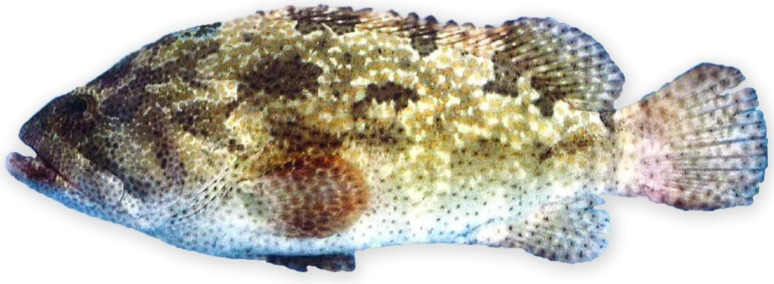
Brown marbled grouper (*Epinephelus fuscoguttatus*) is a big apex predator from the coral reefs of the Indo-Pacific region. (Photo provided by Agriculture, Fisheries and Conservation Department, the Government of the Hong Kong SAR).

In Hong Kong and other coastal cities of southern China, ciguatera was first reported in the late 1980s [6] and the 1990s [7], respectively. With the growing demand for live coral reef fishes and the import of certain fish species from new fishing grounds [8], the incidence of ciguatera has generally increased, and groupers have become the most important cause of ciguatera [7,9]. Brown marbled grouper (*Epinephelus fuscoguttatus*) (Figure 1), also known as flowery cod in Australia and tiger grouper in Hong Kong, was responsible for several large outbreaks of ciguatera in the region [7,9,10]. All of the published case series are reviewed to characterize the types, severity and chronicity of ciguatera symptoms after the consumption of brown marbled grouper.

**Table 1:** Published case series of ciguatera after consumption of brown marbled grouper.

Reference	Sex	Age (years old)	Latent period, clinical features (prevalence or number of subjects affected), treatments and the outcomes
[11] ^a^	54M78F	43.0	0.3–5 h (median 3.8 h).
			GIS—AP/D (67%), N/V (53%), AP (37%).
			NS— ↓ sensation in hands/feet (78%), paresthesia of perioral area/throat (71%), pruritus/formication (69%), myalgia (65%), arthralgia (64%), reversal of hot-cold sensation (36%), numbness of face (4%), ↓ hearing (3%), ↓ vision (2%), post-urination urethral pain (2%), polymyositis (14%).
			CVS—sinus bradycardia (14%), AE (5%), BP < 90/60 mmHg (4%), VE (3%), PR interval >0.2 s (1%).
			Treatments—i.v. dopamine for hypotension.
[12] ^b^	36M27F	43.2 (23–70)	0.5–24 h.
			GIS—AP (84%), D (79%), N/V (78%).
			NS—knee/muscle pain (87%), dizziness/fatigue (82%), paresthesia of 4 limbs (65%), pruritus/formication (62%), reversal of hot-cold sensation (54%), numbness of lips/tongue/throat (33%), altered vision (14%).
			CVS—arrhythmias ^c^ (41%), palpitations/chest tightness/ dyspnea (40%), hypotension/cold sweats (24%).
			Outcomes—GIS/CVS symptoms subsided in 7–14 days. Follow-up for up to 9 months—paresthesia lasted 14–28 days (*n* = 35), 35–56 days (*n* = 18), 56–84 days (*n* = 7) or 180 days (*n* = 2); relapse of symptoms after eating seafood (*n* = 5), drinking alcohol (*n* = 3) or eating peanuts (*n* = 2).
[13] ^d^	45M14F	(3–63)	GIS—0.5–5 h, AP (93%) lasted 1–3 days, D (93%), N (85%), V (85%), hiccup (7%) for 2–7 days, retching (5%) for 2–5 days.
			NS—immediate: numbness of lips (5%); 0.5–5 h to 2–3 days: dizziness (93%), fatigue (93%); 2–3 days: formication or itching (49%), reversal of hot-cold sensation (56%), knee pain/lower limb muscle weakness (20%) lasted 7–14 days.
			CVS—8–16 h, sinus bradycardia (49%) lasted 3–7 days, BP < 90/60 mmHg (44%) lasted 3–7 days, chest tightness (49%), ST segment changes (10%), PR interval >0.2 s (7%), syncope (2%). Concomitant alcohol consumption (*n* = 23) carried a higher risk of bradycardia (78% *vs.* 19%), hypotension (48% *vs.* 14%) and altered skin sensation (96% *vs.* 42%) compared with non-drinkers (*n* = 36). Plasma K < 3.5 mmol/L (7%).
			Treatments—8 subjects treated by i.v. mannitol within 24–72 h; 25 subjects with bradycardia treated by atropine for 3–6 days; 16 subjects with hypotension treated by i.v. dopamine for 3–6 days.
			Outcomes—stayed in hospital for 5–13 days (mean 9.5 days); all were well 3 months later.
[14] ^e^	17M27F	(11–64)	GIS—2–12 h, N (61%), V (61%), AP (61%) lasting 1–2 days, D (61%) lasting 1–2 days.
			NS—2–12 h: knee/calf muscle pain (52%), dizziness (45%), fatigue (45%), numbness of lips/tongue (45%); 2–3 days: hyperalgesia of lower limbs (41%), reversal of hot-cold sensation (18%) for 14–21 days, pruritus or formication (11%).
			CVS—sinus bradycardia (41%) lasting 2–3 days, ST segment changes (11%), systolic BP <90 mmHg (9%). Plasma K < 3.5 mmol/L (50%).
			Outcomes—muscle pain subsiding in 1–2 days, numbness of lips and weakness of 4 limbs disappeared after 7 days; a 11-year old child with dizziness and malaise stayed in hospital for 14 days, 43 other patients stayed for <7 days.
[15] ^f^	29M/F	(9–66)	1st outbreak (*n* = 6)—all 6 subjects had N, V, D, dizziness and numbness of mouth, lips, throat, hands and feet; 2 subjects with larger consumption also had shock, bradycardia, pruritus and reversal of hot-cold sensation.
			2nd outbreak (*n* = 11)—all 11 subjects had similar symptoms as the subjects in the 1st outbreak; 2 subjects also developed shock and coma.
			3rd outbreak (*n* = 12)—all 4 subjects had AP, D and knee pain; they shared the remaining fish with 8 other subjects at dinner the next day; in addition to V and D, all 4 subjects had reversal of hot-cold sensation.
			In total, 29 subjects had GIS; 10 subjects had reversal of hot-cold sensation, pruritus and numbness for 7–10 days, 16 subjects required hospital admission. The latent period was 2–3 h.

Age as mean (range); latent period = time between ingestion and onset of first symptoms; CVS = cardiovascular system; AE = frequent atrial ectopics; VE = frequent ventricular ectopics; GIS = gastrointestinal system; AP = abdominal pain; D = diarrhea; N = nausea; V = vomiting; NS = neurological system; dizziness could be a symptom of the CVS and NS; sweating could be a NS symptom, while cold, sweaty skin could be seen in shock. ^a^ Out of >200 subjects with ciguatera after eating brown marbled grouper at a banquet; latent period (mean ± SD)—paresthesia of perioral area/throat (1.4 ± 0.8 h); ↓ sensation in hands/feet (2.3 ± 0.9 days); pruritus/formication (2.8 ± 1.3 days); reversal of hot-cold sensation (3.6 ± 1.7 days); ^b^ Out of >200 subjects with ciguatera after eating brown marbled grouper at banquets on two consecutive days; latent period/duration of symptom—AP (4.3 ± 2.1 h, 1.8 ± 1.0 days); N/V (5.1 ± 2.9 h, 2.1 ± 1.5 days); D (8.1 ± 4.1 h, 2.7 ± 1.7 days); hypotension/cold sweats (7.6 ± 3.2 h, 3.7 ± 2.4 days); arrhythmias (11.9 ± 5.3 h, 4.9 ± 3.0 days); knee/muscle pain (16.9 ± 6.8 h, 8.1 ± 4.2 days); paresthesia of 4 limbs (2.0 ± 0.7 days, 29.1 ± 8.7 days); pruritus/formication (2.6 ± 1.1 days, 25.9 ± 8.1 days); reversal of hot-cold sensation (3.0 ± 1.5 days, 23.2 ± 7.8 days); ^c^ The nature of arrhythmias was not specified; ^d^ Fifty-nine subjects with ciguatera after eating brown marbled grouper (>7 kg) at lunch or dinner in a restaurant on the same day; ^e^ Out of >100 subjects with ciguatera after eating brown marbled grouper at a banquet; ^f^ From two families and one family and friends with ciguatera after eating brown marbled grouper bought in the same shop at home dinners; the left-over samples were tested positive for CTX using Cigua-Check® (immune-reactive test kit, ToxiTec, Honolulu, HI, USA) and a mouse bioassay (0.11 MU/g).

## 2. Published Case Series of Ciguatera after Consumption of Brown Marbled Grouper

To identify relevant papers in indexed journals and Chinese medical journals, a search of the Medline (1980 to 16 May 2014) and China Journal Jet (1994 to May 2014) was performed, using ciguatera, ciguatoxins, *E. fuscoguttatus* and brown marbled grouper as the search terms. Case series of ciguatera lacking separate details for subjects with the consumption of brown marbled grouper were excluded.

Five published case series of ciguatera after consumption of brown marbled grouper were identified (Table 1). In Guangdong Province, China, four hospitals in Zhongshan [11,12], Shantou [13] and Foshan [14] each treated 44–132 subjects in August and November, 2004. Three of these case series [11,12,14] were from large outbreaks affecting over 100–200 subjects who had eaten this reef fish served at banquets. In Fujian Province, China, a hospital in Xiamen [15] treated 29 subjects in February, 2005. All five case series were brief, with variable amounts of details about the clinical progress of affected subjects, investigations, treatments given and the outcomes. The amounts of fish consumed were not mentioned. The size of the fish involved (>7 kg) was mentioned in only one report [13]. Where the latent period between fish consumption and appearance of first symptoms and the duration of illnesses were described, such information was presented in Table 1. The signs and symptoms of affected individuals were categorized as gastrointestinal, neurological and cardiovascular [1,16].

As can be seen in Table 1, affected subjects generally developed a combination of gastrointestinal, neurological and, less commonly, cardiovascular symptoms. Gastrointestinal symptoms (e.g., nausea, vomiting, abdominal pain and diarrhea) occurred in the early stages (within 4–8 h of fish consumption), but generally subsided in 1–2 days [12]. Neurological symptoms occurred early (e.g., paresthesia/numbness of lips, tongue and throat) or late (e.g., paresthesia of four limbs, reversal of hot-cold sensation, myalgia, muscle weakness, arthralgia, pruritus, formication and fatigue), *i.e.*, within hours or days of fish ingestion [11,12,13]. Neurological symptoms (e.g., paresthesia of four limbs) could last for weeks or months [12]. Sinus bradycardia and hypotension occurred early (within hours of fish ingestion) and could be severe and prolonged, requiring treatments with atropine and dopamine for 3–6 days [13]. Other cardiovascular and neurological features included palpitations, atrial ectopics, ventricular ectopics, dyspnea, chest tightness, PR interval >0.2 s, ST segment changes, polymyositis and coma.

Variations in the types, severity and chronicity of symptoms were also obvious within and between the case series (Table 1). For example, polymyositis [11] and coma [15] were reported by one case series each, but not by others. The incidence of sinus bradycardia and hypotension varied between 14%–49% and 4%–44%, respectively [11,12,13,14]. Both the duration and severity of sinus bradycardia and hypotension and, hence, the need for atropine and dopamine, also differed [12,13,14]. Paresthesia of four limbs persisted much longer in some subjects than in others [12].

It was also worth noting that concomitant alcohol consumption increased the incidence of neurological and cardiovascular symptoms [13], and the subsequent ingestion of seafood, alcohol or peanuts could trigger the relapses of symptoms in some subjects [12].

## 3. Discussion

Many species and many families of coral reef fishes are commonly involved in ciguatera worldwide, and the predominant ciguatoxic fishes vary with the geographical region [4]. The larger carnivores, such as moray eels, snappers, groupers, Spanish mackerels and barracuda, should be avoided, as they are likely to be more toxic [4,16]. In Hong Kong and other coastal cities of southern China, groupers become the most important cause of ciguatera, and brown marbled grouper was responsible for several large outbreaks [7,9,10,11,12,13,14,15].

Brown marbled grouper is widely distributed in shallow coral reefs and rocky bottoms of the warm Indo-Pacific region. Like humphead wrasse [5], it is a much sought-after, high-valued species in the live reef food-fish trade. It can grow to a maximum size of >100 cm total length, weighing >17.1 kg [17].

The knowledge of ciguatera and the species and size of fish to avoid [8] may affect the decisions about eating a potentially ciguatoxic fish. Despite the risk of toxicity, brown marbled grouper is commonly consumed. In taking their chances, the public adopts a risk-based approach by avoiding larger fish. However, the maximum fish size that is regarded as relatively safe varies across the Pacific region. In Australia [18], large fish are considered unpalatable due to concerns of possible ciguatera, and commercial fishers have difficulty in selling fish weighing >6 kg. In New Caledonia [19], the ciguatoxic potential of this grouper is considered medium; the size (total length) threshold above which the risk of ciguatera significantly increases is 40 cm or 1.0 kg based on its known length-weight relationships [17]. In Hong Kong [20], fish weighing <1.8 kg are more popular, because the risk of toxicity is assumed to be lower and plate-sized fish are considered the best for consumption.

As expected of an apex predator from coral reefs of the Indo-Pacific region [2], brown marbled grouper can cause ciguatera, especially if larger fish from high-risk areas and greater amounts (particularly the viscera) are consumed. In Pacific island countries and territories [21], brown marbled grouper was known to cause several outbreaks of ciguatera in Kiribati, the Fiji Islands and Tokelau. In Australia [22], it was responsible for five outbreaks (14 cases) in Queensland in 1965–1984. In Cairns, North Queensland [23], a woman developed ciguatera after eating a brown marbled grouper; this fish was found to contain Pacific CTX-1 (P-CTX-1), P-CTX-2 and P-CTX-3. In Queensland [23], a brown marbled grouper implicated in a previous outbreak was also found to contain P-CTX-1 (0.3 µg/kg fish flesh), P-CTX-2 and P-CTX-3. In Okinawa, Japan [24], this fish accounted for two ciguatera outbreaks (three cases) in 1999. Out of 24 brown marbled grouper samples collected from Okinawa market, five (21%) were found to be ciguatoxic using a mouse bioassay. There was a parallel increase in toxicity and body weight; all five toxic samples were from fish >70 cm total length, weighing >13.3 kg. In Hong Kong [25], brown marbled grouper >5 kg was responsible for most of the 12 ciguatera outbreaks (40 cases) in early May, 1998.

Brown marbled grouper has long been known to cause ciguatera in the Pacific region [21] and was responsible for several large outbreaks in recent years [7,9,10]. Surprisingly, there is a lack of clinical reports, especially individual data, to characterize the types, severity and chronicity of ciguatera symptoms associated with its consumption and acute exposure to P-CTX. Hospital-based case studies are preferred, since information on symptoms obtained by phone interviews might fail to identify bradycardia, hypotension and abnormal test results [5].

Although the same reef fish species containing P-CTX was involved in the five case series, there were considerable inter-individual variations in the types, severity and chronicity of ciguatera symptoms (Table 1). Large variations in symptoms have also been observed among individuals with ciguatera after consumption of humphead wrasse [5]. The severity and duration of symptoms are typically dose-dependent, with the ingestion of a large quantity and the CTX-rich parts (head, viscera) causing more severe poisoning and prolonged illness [5,26,27]. Ingestion of fish head and viscera is a strong risk factor for the severe form of ciguatera, *i.e.*, more severe symptoms in the acute phase and persistence of symptoms 15 days after the onset [27]. Chronic alcohol use and concomitant alcohol ingestion also increase the risk of severe ciguatera [28,29]. Concomitant alcohol consumption was associated with a much higher risk of developing bradycardia, hypotension and altered skin sensation (Table 1). Repeated exposures to CTX also result in a more severe illness [28,29]. Increasing age and body weight, presumably associated with greater lifetime exposures and greater capacity for CTX storage [2], are related to the severity and duration of symptoms [30]. There is also considerable evidence of individual susceptibility, as the incubation period, symptoms and attack rates are highly variable, even among subjects who have eaten the same fish (Table 1) [5,22].

Epidemiological, hazard and clinical characterization of ciguatera has been limited by under-reporting, incomplete information on fish consumption and symptoms and a lack of fish remnants for species identification and CTX quantification. CTX, even from the same region, exist in multiple forms with differing toxicities [2], and symptoms are CTX-specific or fish-specific [4]. Therefore, regional reports, as well as individual data for major ciguatoxic fish species should also be analyzed. The present review of five published case series helps characterize the ciguatera caused by consumption of brown marbled grouper. Affected subjects generally developed a combination of gastrointestinal, neurological and, less commonly, cardiovascular symptoms (see Table 1). Gastrointestinal symptoms occurred early and generally subsided in 1–2 days. Some neurological symptoms (e.g., paresthesia of four limbs) could last for weeks or months. Sinus bradycardia and hypotension occurred early, but could be severe and prolonged, necessitating the timely use of intravenous fluids, atropine and dopamine [5]. Other cardiovascular and neurological features included atrial ectopics, ventricular ectopics, dyspnea, chest tightness, PR interval >0.2 s, ST segment changes, polymyositis and coma.

This review also reflects well the potential of an apex predator from coral reefs of the Indo-Pacific region to cause large ciguatera outbreaks each affecting >100–200 subjects when such a fish is served at banquets [11,12,14]. To prevent ciguatera outbreaks, the public should be reminded to avoid eating large coral reef fishes, especially the CTX-rich fish parts—head, viscera, roe and skin [4,8]. They should realize that consumption of the high-risk fish (especially the CTX-rich parts and together with alcohol use) and repeated CTX exposures will result in more severe and chronic illness. Regulatory measures should be considered, e.g., bans on high-risk fish and restrictions on fish from high-risk areas [1]. This will be useful to monitor the prevalence of ciguatoxic fish in the market and characterize the size threshold for potential toxicity, so that plans for the cost-effective management of ciguatera risk can be planned [19].

## 4. Conclusions

Brown marbled grouper is an apex predator from coral reefs of the Indo-Pacific region. Consumption of this grouper was responsible for several large outbreaks of ciguatera in the region. Affected individuals generally developed a combination of gastrointestinal, neurological and, less commonly, cardiovascular symptoms. Gastrointestinal symptoms were self-limiting, but some neurological symptoms (e.g., paresthesia of four limbs) could last for weeks or months. Sinus bradycardia and hypotension occurred early, but could be severe and prolonged, necessitating the timely use of intravenous fluids, atropine and dopamine. To prevent ciguatera outbreaks, the public should be reminded to avoid eating large coral reef fishes, especially the CTX-rich fish parts—head, viscera, roe and skin. They should realize that consumption of the high-risk fish (especially the CTX-rich parts and together with alcohol use) and repeated CTX exposures will result in more severe and chronic illness. 
